# Análisis espacial de la hipertensión arterial en adultos peruanos, 2022

**DOI:** 10.47487/apcyccv.v4i2.296

**Published:** 2023-06-30

**Authors:** Akram Hernández-Vásquez, Brenda Noemí Carrillo Morote, Victoria del Carmen Azurin Gonzales, Efraín Y. Turpo Cayo, Diego Azañedo

**Affiliations:** 1 Centro de Excelencia en Investigaciones Económicas y Sociales en Salud, Vicerrectorado de Investigación, Universidad San Ignacio de Loyola, Lima, Perú Universidad San Ignacio de Loyola Centro de Excelencia en Investigaciones Económicas y Sociales en Salud Vicerrectorado de Investigación Universidad San Ignacio de Loyola Lima Peru; 2 Facultad de Ciencias de la Salud, Universidad Científica del Sur, Lima, Perú. Universidad Científica del Sur Facultad de Ciencias de la Salud Universidad Científica del Sur Lima Peru; 3 Investigadora independiente, Lima, Perú. Investigadora independiente Lima Perú; 4 Universidad Nacional Agraria La Molina, Lima, Perú. Universidad Nacional Agraria La Molina Universidad Nacional Agraria La Molina Lima Peru; 5 Universidad Científica del Sur, Lima, Perú. Universidad Científica del Sur Universidad Científica del Sur Lima Peru

**Keywords:** Análisis Espacial, Hipertensión, Encuestas Epidemiológicas, Per*ú*, Spatial Analysis, Hypertension, Health Surveys, Peru

## Abstract

**Objetivo.:**

Realizar un análisis espacial de la hipertensión arterial en la población adulta peruana, para identificar patrones geográficos con una mayor concentración de casos.

**Materiales y métodos.:**

Se realizó un análisis espacial utilizando datos de la encuesta demográfica y de salud familiar (ENDES) 2022. Se incluyó a una muestra de 29 422 adultos y se utilizó el índice global de Moran además del análisis de puntos calientes para evaluar la autocorrelación espacial y la concentración de casos.

**Resultados.:**

La prevalencia de hipertensión arterial estandarizada por edad fue del 19,2%. Se observaron conglomerados con una alta concentración de hipertensión arterial en departamentos de la costa peruana como Tumbes, Piura, Lambayeque, La Libertad, Ancash y Lima, así como en las regiones de la sierra norte. También se encontraron conglomerados en las regiones de Loreto y Madre de Dios en la selva peruana.

**Conclusiones.:**

Este estudio reveló patrones geográficos de hipertensión arterial en el Perú, con una mayor concentración de casos en la costa peruana y algunas regiones de la sierra y selva. Estos hallazgos resaltan la necesidad de desarrollar estrategias de prevención y control de la enfermedad, especialmente en las áreas identificadas como conglomerados de alta prevalencia.

## Abstract (en)

**Objectives.:**

To perform a spatial analysis of arterial hypertension in the Peruvian adult population to identify geographic patterns with a higher concentration of cases.

**Materials and methods:**

A spatial analysis was conducted using data from the Demographic and Family Health Survey (ENDES) 2022. A sample of 29,422 adults was included, and the global Moran’s index and Getis-Ord Gi* analysis were used to evaluate spatial autocorrelation and cluster concentration.

**Results:**

The age-standardized prevalence of arterial hypertension was 19.2%. Clusters with a high concentration of arterial hypertension were observed in departments along the Peruvian coast such as Tumbes, Piura, Lambayeque, La Libertad, Ancash, and Lima, as well as in the northern regions of the Highlands. Clusters were also found in the regions of Loreto and Madre de Dios in the Peruvian jungle.

**Conclusions:**

This study revealed geographic patterns of arterial hypertension in Peru, with a higher concentration of cases along the Peruvian coast and in certain regions of the Highlands and Jungle. These findings highlight the need to develop strategies for the prevention and control of the disease, especially in the areas identified as high-prevalence clusters.

## Introducción

La hipertensión arterial es una enfermedad crónica de naturaleza multifactorial y de gran carga de enfermedad que afecta aproximadamente a 1280 millones de personas en todo el mundo ^1^^,^^2^. Según el informe de la Organización Mundial de la Salud (OMS) del 2022, alrededor del 22% de la población mundial presentó hipertensión arterial; asimismo, la prevalencia en seis países de América Latina (Argentina, Brasil, Chile, Colombia, Perú y Uruguay), alcanzó un 32,3% ^3^, siendo el 68 y 32% en áreas urbanas y rurales de estos países, respectivamente.

El consumo excesivo de alcohol, tabaquismo, sedentarismo, dietas poco saludables y estrés crónico son algunos de los factores de riesgo conocidos para la hipertensión arterial; asimismo, algunos reportes muestran que la enfermedad está relacionada con la edad, el sexo y el área de residencia (urbana o rural) ^(^^2^^,^^4^^,^^5^. Una revisión sistemática con metaanálisis publicada recientemente mostró que hubo una mayor prevalencia de hipertensión arterial en las áreas urbanas (30,5 %) en comparación con las áreas rurales (27,9%) a nivel mundial ^6^. Estos resultados enfatizan la necesidad de medidas preventivas y de control de la enfermedad, especialmente en las áreas urbanas donde los hábitos y estilos de vida pueden producir un mayor riesgo de desarrollarla ^7^. Asimismo, las prevalencias elevadas de hipertensión en áreas rurales, donde los niveles de concientización, tratamiento y control de la hipertensión siguen siendo considerablemente más bajos en comparación con las áreas urbanas, nos muestran que también es necesario dirigir estrategias hacia este grupo de la población, para evitar el incremento en la frecuencia de hipertensión ^6^. Es importante mencionar que, si no se realiza un control adecuado de la enfermedad, esto podría ocasionar una mayor incidencia de accidentes cerebrovasculares, infartos de miocardio, enfermedad renal crónica e incluso la muerte, entre la población ^8^.

 En el año 2022 en el Perú se realizó un informe nacional sobre las enfermedades no transmisibles (ENT) donde se reportó que alrededor de 5,5 millones de personas mayores de 15 años presentaron hipertensión arterial, lo que es equivalente al 22,1% de este grupo poblacional ^9^. En cuanto a la distribución geográfica del país, la región con mayor prevalencia de hipertensión arterial fue la costa (24,4%), seguida de la sierra (18,7%) y la selva (17,2%). Sin embargo, la mayor prevalencia se reportó en las áreas urbanas del país, con un 17,2 % en comparación con el 11,9% en el área rural. En cambio, a nivel departamental, las mayores prevalencias se registraron en Lima (27,1%), Loreto (24,0%), y Tumbes (23,2%), mientras que los departamentos con menor prevalencia fueron Apurímac (12,9%), y Ucayali (9,4%) ^10^. En ese sentido, se puede mencionar que los resultados que se muestran con respecto a la prevalencia de la hipertensión arterial dependen mucho de la etapa de urbanización a nivel del país y del desarrollo socioeconómico. De modo que se requiere de un análisis más detallado de la distribución espacial de la hipertensión en el Perú, para poder focalizar las intervenciones y disminuir los efectos nocivos de esta enfermedad, la cual es un factor de riesgo modificable de mortalidad cardiovascular ^8^.

El objetivo del presente estudio fue realizar un análisis espacial de la hipertensión arterial en la población peruana, con el fin de identificar patrones geográficos con mayor concentración de casos. Los resultados obtenidos permitirán una mejor comprensión de la distribución espacial de la hipertensión que sirva de base para el desarrollo de estrategias de prevención en las áreas identificadas.

## Materiales y métodos

### Diseño y población de estudio

Se realizó un análisis espacial utilizando los datos georreferenciados de la encuesta demográfica y de salud familiar (ENDES) 2022. El Instituto Nacional de Estadística e Informática del Perú (INEI) realiza esta encuesta año a año para recopilar datos sobre los hogares, las mujeres en edad reproductiva y sus hijos menores de cinco años. Además, la ENDES incluye un cuestionario de salud para un miembro del hogar de 15 años o más y para todos los menores de 12 años, con el objetivo de proporcionar información sobre factores de riesgo modificables, prevalencia y acceso a tratamiento de enfermedades no transmisibles, así como acceso a servicios de salud, entre otros ^10^.

### Muestreo y selección de participantes

El muestreo de la ENDES 2022 fue probabilístico, bietápico, estratificado e independiente. La población objetivo la formaron los residentes habituales de viviendas particulares, incluyendo aquellos que no eran residentes, pero que pernoctaron en la vivienda la noche anterior a la encuesta. El informe final y ficha técnica de la encuesta contienen más detalles metodológicos sobre la ENDES 2022 ^10^. El marco muestral se basó en la información estadística y cartográfica de los Censos Nacionales XI de Población y VI de Vivienda del año 2017, así como en el material cartográfico elaborado para la ejecución de la ENDES 2022. El método utilizado para la recolección de datos fue realizado mediante entrevista directa en las viviendas seleccionadas, realizada por personal debidamente capacitado por el INEI para el recojo de los datos.

En el presente estudio se incluyó a una submuestra de encuestados de 18 años o más que participaron en el cuestionario salud de la ENDES. En total, se incluyeron 29 422 adultos después de excluir a los menores de 18 años y a los registros con datos incompletos en la variable de interés.

### Variables de estudio

La variable principal de estudio fue la presencia de hipertensión arterial. Si la presión arterial media se encontró por encima de 140 mmHg en la presión arterial sistólica y/o 90 mmHg en la diastólica ^11^, la variable se categorizó como 1 (presencia de hipertensión arterial), en caso contrario se codificó como 0 (sin hipertensión arterial). Para determinar el valor de la presión arterial se realizaron dos mediciones de la presión arterial sistólica (PAS) y diastólica (PAD), y se utilizó el valor promedio de la PAS y el valor promedio de la PAD ^10^.

La medición de la presión arterial se realizó con un tensiómetro automático de la marca OMRON y modelo HEM-7113, que tiene un rango de medición de 0 a 299 mmHg y precisión de ±3 mmHg ^10^. Según la contextura del entrevistado se emplearon dos tipos de brazaletes, uno estándar (220 a 320 mm) y otro para brazos de mayor volumen (320 a 420 mm) ^10^.

Las variables utilizadas para el análisis espacial fueron la latitud y longitud del conglomerado en el que se geolocalizaba la vivienda del entrevistado. Estas variables se recopilaron mediante el sistema de posicionamiento global (GPS, por sus siglas en inglés) incluido en una tableta, colocada a un metro de la puerta principal de la vivienda. Más información sobre la medición del proceso de geolocalización en la ENDES 2022 se puede encontrar en el manual de la encuesta ^12^.

Finalmente, se incluyeron las siguientes variables para caracterizar a la población de estudio: sexo (hombres/mujeres); grupo etario (18-29/30-59/60 o más); nivel educativo (hasta primaria/secundaria/superior); autoidentificación étnica (no nativo, nativo, Afroperuano); casado o conviviente en los últimos 12 meses (sí/no); estado nutricional (hasta normal/sobrepeso/obesidad); diagnóstico previo de diabetes (no/sí); fumador actual (no/sí); bebedor actual (no/sí); índice de riqueza (más pobre/pobre/medio/rico/más rico); región de residencia (Lima Metropolitana, resto de la costa/sierra/selva), y área de residencia (urbana/rural).

### Análisis estadístico

Los datos se procesaron y analizaron utilizando Stata versión 17 (StataCorp, College Station, TX, EE. UU.). En primer lugar se realizó un análisis descriptivo y se estimó la prevalencia de hipertensión arterial según las características de los participantes incluidos. Asimismo, se estimó la prevalencia de hipertensión arterial estandarizada por edad utilizando el estándar de población de la OMS ^13^. Se aplicó la prueba chi cuadrado para evaluar diferencias entre la variable de interés y las características evaluadas. En segundo lugar se realizó un análisis espacial siguiendo la metodología empleada en artículos anteriores ^14^.

El análisis espacial incluyó una evaluación de la autocorrelación espacial (índice global de Moran y Anselin Local Moran’s I) y un análisis de puntos calientes (estadística Gi* de Getis-Ord) a nivel distrital (análisis exploratorio), utilizando el *software* de sistema de información geográfica ArcGIS Desktop versión 10.5 (ESRI Inc., Redlands, CA, EE. UU.). En el presente estudio el índice global de Moran evaluó el patrón general y la tendencia de los casos con hipertensión arterial para determinar si estaban agrupados, dispersos o distribuidos aleatoriamente. El índice global de Moran varía entre -1 y +1, donde un valor positivo indica agrupación espacial, un valor de 0 indica un patrón distribuido aleatoriamente, y valores negativos indican un patrón disperso.

### Consideraciones éticas

El estudio no requirió la aprobación de un comité de ética ya que se trató de un análisis de datos secundarios que son de dominio público y no permiten identificar a los participantes incluidos.

## Resultados

Se incluyeron 29 422 adultos peruanos. Se observó una mayor frecuencia de participantes mujeres (52,1%), con edades comprendidas entre 30 y 59 años (54,5%), que se autoidentificaban como no nativos (58,7%), casados o convivientes en los últimos 12 meses (65,7%), residentes en Lima Metropolitana (37,4%), y en áreas urbanas (81,8%) (Tabla 1).

**Tabla 1:** Descripción de las características de los encuestados incluidos (n=29422)

Características	n		%*
Sexo			
Hombre	12 707		47,9
Mujer	16 715		52,1
Grupo etario			
18-29 años	8378		26,9
30-59 años	16 825		54,5
60 o más años	4219		18,6
Nivel educativo			
Hasta primaria	7946		22,9
Secundaria	12665		43,3
Superior	8811		33,8
Autoidentificación étnica**			
No nativo	13 895		58,7
Nativo	11 048		29,3
Afroperuano	3055		12,0
Casado o conviviente en los últimos 12 meses**			
Sí	20 782		65,7
No	8638		34,3
Estado nutricional			
Hasta normal	10 717		34,7
Sobrepeso	11 394		38,6
Obesidad	7311		26,6
Diagnóstico previo de diabetes			
No	28 283		94,6
Sí	1139		5,4
Fumador actual			
No	26 617		90,0
Sí	2805		10,0
Bebedor actual			
No	19 330		61,9
Sí	10 092		38,1
Quintil de riqueza			
Más pobre	9498		18,5
Pobre	7554		20,3
Medio	5498		21,3
Rico	3982		20,5
Más rico	2890		19,5
Región de residencia			
Lima Metropolitana	3426		37,4
Resto de costa	8246		25,9
Sierra	10660		24,2
Selva	7090		12,5
Área de residencia			
Urbana	18 998		81,8
Rural	10 424		18,2

*La estimación incluyó el factor de ponderación y el diseño muestral de la ENDES 2022.

** Variable contiene valores perdidos.

La prevalencia de hipertensión arterial estandarizada por edad fue del 19,2% (IC 95%: 18,3-20,1). Se encontró una mayor prevalencia de hipertensión arterial en hombres (21,6%), adultos de 60 o más años (35,3%), personas con nivel educativo hasta primaria (21,4%), no nativos (18,0%), personas con obesidad (27,2%) o diagnóstico previo de diabetes (33,7%), en el quintil de riqueza más rico (21,7%), residentes en Lima Metropolitana (22,1%) y en áreas urbanas (18,2%) (Tabla 2). Las menores prevalencias se encontraron en adultos de 18 a 29 años (5,3%), con un estado nutricional hasta normal (11,9%), y residentes en la región selva (12,1%) (Tabla 2).

**Tabla 2:** Prevalencia de hipertensión arterial según características de la población adulta peruana

Características	Hipertensión arterial				
	No (n=25 821) % (IC 95%)		Sí (n=3601) % (IC 95%)		Valor de p*
Total	82,8 (82,0-83,6)		17,2 (16,4-18,0)		
Total estandarizado por edad**	80,8 (79,9-81,7)		19,2 (18,3-20,1)		
Sexo					
Hombre	78,4 (77,2-79,7)		21,6 (20,3-22,8)		<0,001
Mujer	86,8 (85,8-87,8)		13,2 (12,2-14,2)		
Grupo etario					
18-29 años	94,7 (93,7-95,5)		5,3 (4,5-6,3)		<0,001
30-59 años	83,1 (82,0-84,2)		16,9 (15,8-18,0)		
60 o más años	64,7 (62,3-67,1)		35,3 (32,9-37,7)		
Nivel educativo					
Hasta primaria	78,6 (77,0-80,2)		21,4 (19,8-23,0)		<0,001
Secundaria	83,5 (82,2-84,7)		16,5 (15,3-17,8)		
Superior	84,8 (83,5-86,0)		15,2 (14,0-16,5)		
Autoidentificación étnica					
No nativo	82,0 (80,9-83,1)		18,0 (16,9-19,1)		0,001
Nativo	85,2 (83,9-86,4)		14,8 (13,6-16,1)		
Afroperuano	82,8 (80,6-84,9)		17,2 (15,1-19,4)		
Casado o conviviente en los últimos 12 meses					
Sí	84,0 (83,0-84,9)		16,0 (15,1-17,0)		<0,001
No	80,6 (79,0-82,0)		19,4 (18,0-21,0)		
Estado nutricional					
Hasta normal	88,1 (86,9-89,2)		11,9 (10,8-13,1)		<0,001
Sobrepeso	84,9 (83,7-86,1)		15,1 (13,9-16,3)		
Obesidad	72,8 (71,1-74,4)		27,2 (25,6-28,9)		
Diagnóstico previo de diabetes					
No	83,8 (83,0-84,5)		16,2 (15,5-17,0)		<0,001
Sí	66,3 (61,6-70,7)		33,7 (29,3-38,4)		
Fumador actual					
No	83,0 (82,2-83,9)		17,0 (16,1-17,8)		0,097
Sí	80,9 (78,3-83,3)		19,1 (16,7-21,7)		
Bebedor actual					
No	82,4 (81,4-83,4)		17,6 (16,6-18,6)		0,205
Sí	83,4 (82,1-84,6)		16,6 (15,4-17,9)		
Quintil de riqueza					
Más pobre	87,3 (86,2-88,4)		12,7 (11,6-13,8)		<0,001
Pobre	85,5 (84,1-86,8)		14,5 (13,2-15,9)		
Medio	82,2 (80,4-83,8)		17,8 (16,2-19,6)		
Rico	81,0 (79,1-82,8)		19,0 (17,2-20,9)		
Más rico	78,3 (76,0-80,4)		21,7 (19,6-24,0)		
Región de residencia					
Lima Metropolitana	77,9 (76,1-79,6)		22,1 (20,4-23,9)		<0,001
Resto de costa	83,7 (82,5-84,8)		16,3 (15,2-17,5)		
Sierra	86,9 (85,9-87,8)		13,1 (12,2-14,1)		
Selva	87,9 (86,7-89,0)		12,1 (11,0-13,3)		
Área de residencia					
Urbana	81,8 (80,9-82,8)		18,2 (17,2-19,1)		<0,001
Rural	87,2 (86,2-88,1)		12,8 (11,9-13,8)		

Los datos se muestran como % ponderado de la fila a menos que se indique lo contrario.

Todas las estimaciones incluyeron el factor de ponderación y el diseño muestral de la encuesta demográfica y de salud familiar 2022.

* Valor de p estimado mediante la prueba de chi cuadrado con ajuste de Rao-Scott.

** Prevalencia estandarizada por la población de la Organización Mundial de Salud.

**Figura 1:**
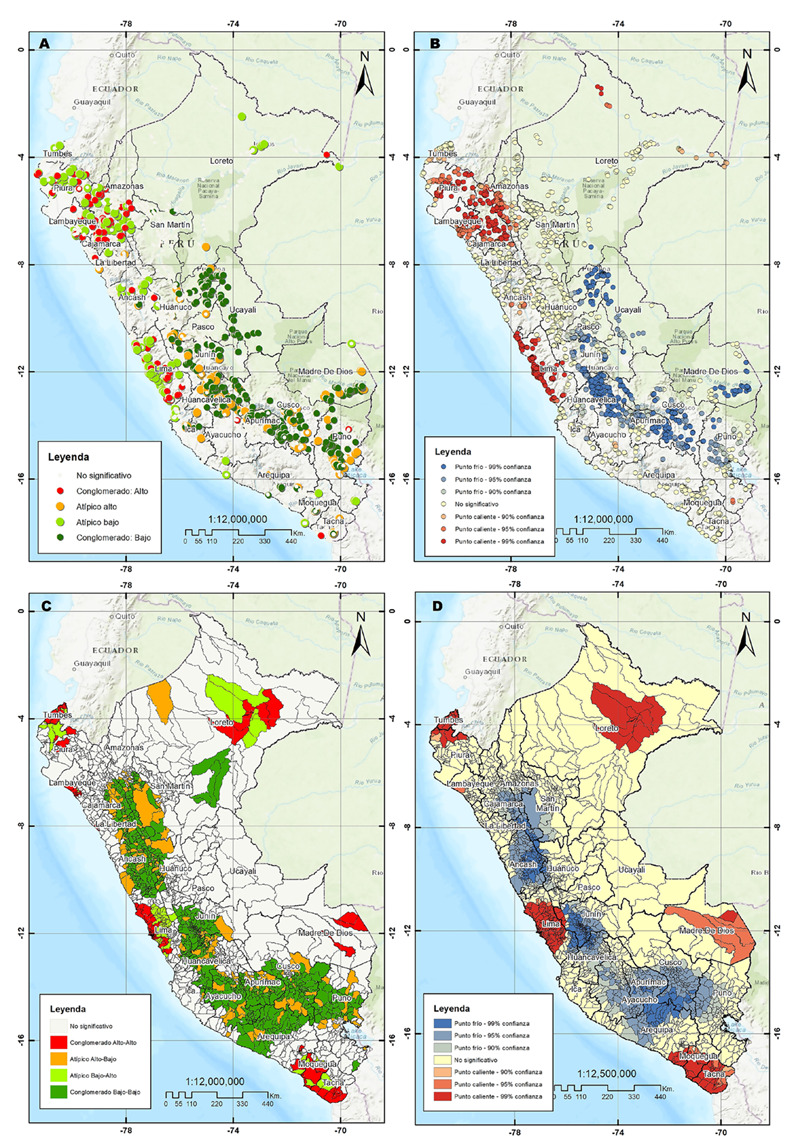
Análisis de la hipertensión arterial en adultos peruanos según la encuesta demográfica y de salud familiar 2022. **A.** Análisis de conglomerados y valores atípicos por análisis de Morán. **B.** Análisis de puntos calientes basado en la estadística Gi* de Getis-Ord. **C.** Análisis de conglomerados y valores atípicos distritales mediante el índice local de Moran. **D.** Análisis de puntos calientes distritales basado en la estadística Gi* de Getis-Ord.

El análisis de autocorrelación espacial identificó conglomerados de participantes con hipertensión arterial (índice global de Moran 0,099587, p< 0,001 y z-score 19,44). Se identificaron conglomerados con una alta concentración de encuestados con hipertensión arterial en los departamentos de la costa peruana como Tumbes, Piura, Lambayeque, La Libertad, Ancash, y Lima, así como en las regiones de la sierra norte como Cajamarca y Amazonas **(**Figura 1A y 1B**)**. El análisis distrital de Moran encontró conglomerados con una alta concentración de participantes con hipertensión arterial rodeados de conglomerados con características similares (conglomerados alto-alto) en regiones de la costa peruana mencionadas previamente, a las que se suman Moquegua y Tacna. También se encontraron conglomerados alto-alto en los departamentos de la selva peruana en Loreto y Madre de Dios **(**Figura 1C y 1D**).** En general, los conglomerados con una baja frecuencia de encuestados con hipertensión arterial rodeados de conglomerados de similares características (conglomerados bajo-bajo) se localizaron en los departamentos de la sierra peruana (Figura 1).

## Discusión

El objetivo del estudio fue realizar un análisis espacial de la hipertensión arterial en la población peruana, con el fin de identificar patrones geográficos con mayor concentración de casos. Se identificó que la prevalencia de hipertensión arterial a nivel nacional fue del 19,2%, resultado similar a lo informado por la OMS que reporta una prevalencia de hipertensión en Perú del 18% en mujeres y 23% en hombres ^15^, aunque un metaanálisis sobre la prevalencia e incidencia de hipertensión determinó que la frecuencia de esta patología oscilaba entre el 20 y 25% ^16^. El Perú se encuentra entre los 10 países con menor prevalencia de personas con hipertensión, a comparación de países como Paraguay (51%), Tuvalu (51%), Republica Dominicana (49%), Jamaica (48%) (15), entre otros. En México se informa que aproximadamente un tercio de su población presenta hipertensión arterial ^17^, mientras que Argelia registra una prevalencia de 31,6% ^18^ y Oriente Medio del 24,4% ^19^.

Según nuestros hallazgos, en Lima Metropolitana y la región de la costa peruana se concentra el mayor número de conglomerados alto-alto de hipertensión arterial. Esto podría deberse a que la costa peruana se caracteriza por tener población mayoritariamente urbana ^20^, donde también se presenta un elevado número de hipertensos a comparación del área rural. Esta concentración de hipertensión en las zonas urbanas puede responder a que las personas son menos activas físicamente y son más propensas a fumar y consumir alcohol ^21^. El conocer la real distribución espacial de una de las enfermedades no transmisibles más prevalentes como la hipertensión arterial, es útil para reconocer puntos estratégicos que permitan focalizar estrategias preventivas inmediatas.

La mayor cantidad de encuestados hipertensos se concentraron en departamentos de la costa peruana como Tumbes, Piura, Lambayeque, La Libertad, Ancash y Lima, esto podría deberse a la mayor concentración de recursos en salud en la costa, por lo cual es más factible desarrollar más tamizajes, y, por lo tanto, captar un mayor número de hipertensos, a diferencia de la sierra peruana, principalmente en entornos rurales donde la población no suele acudir a los centros de salud o no suelen recibir al personal de salud en sus hogares ^16^. Por lo tanto, se obtiene un menor número de encuestados hipertensos, lo cual explicaría porqué en nuestro estudio existen conglomerados bajo-bajo en la sierra peruana a excepción de la sierra norte donde están ubicados los departamentos de Cajamarca y Amazonas, los cuales presentan una elevada densidad poblacional ^22^, al igual que la región Loreto, donde también se ha encontrado conglomerados altos. También se identificaron conglomerados alto-alto en Madre de Dios, lo cual probablemente se deba al rápido desarrollo urbanístico de dicha región tras la construcción de la carretera interoceánica, lo cual contribuye al sedentarismo, malos hábitos alimenticios y obesidad, factores de riesgo conocidos para la hipertensión. Asimismo, otras teorías atribuyen la presencia de esta enfermedad, a la contaminación por mercurio debido a la explotación de la minería informal ^23^^,^^24^, aunque, no se han desarrollado estudios prospectivos confirmando estas hipótesis.

El presente estudio nos brinda una aproximación general para entender la dinámica de la distribución espacial de la hipertensión en el Perú. En ese sentido, es importante que las políticas existentes contra la hipertensión incluyan lineamientos integrales para la prevención, tratamiento y vigilancia epidemiológica de manera efectiva. Estos lineamientos deben poner un enfoque especial en las poblaciones más vulnerables. Es imperativo recalcar que las políticas no solo deben comprender el reconocimiento e intervenciones sobre los factores de riesgo para la hipertensión, sino que es necesario además, que el Estado desarrolle programas que brinden apoyo social, en conjunto con la educación, para que estas políticas logren ser eficaces.

Entre las limitaciones del estudio encontramos, primero, el tipo de diseño, al ser transversal no podemos establecer causalidad, segundo, posibilidad de sesgo debido a que el diagnóstico de hipertensión no sea el indicado por posibles problemas en la técnica de medición o sesgo de información, debido al error u omisión de los encuestadores, y sesgo de deseabilidad social, debido a que los entrevistados pueden alterar sus respuestas para encajar en estándares socialmente aceptados, como el no aceptar el consumo de tabaco y alcohol por ser malos hábitos. Asimismo, el análisis de puntos calientes a nivel distrital son exploratorios considerando la falta de representatividad de la ENDES a nivel de distritos. En cuanto al diagnóstico de hipertensión arterial, considerando que pueden existir diferencias entre la primera y la segunda medición de la presión arterial, sería recomendable contar con una tercera medición en la ENDES, para seguir las recomendaciones aplicadas al diagnóstico de hipertensión arterial en el consultorio ^25^. Aunque las mediciones de la presión arterial se realizaron con un equipo que no figura en las listas de dispositivos automáticos validados para medir la presión arterial ^25^, es importante señalar que, la validación de la precisión de los equipos no es obligatoria antes de su distribución en el mercado y los resultados de las validaciones son específicas para la población analizada y es posible que los equipos no siempre brinden mediciones precisas en individuos de una población tan heterogénea como la peruana. Sin embargo, la ENDES recopila gran cantidad de información nacional y departamental basándose en la información recopilada del cuestionario del hogar y cuestionario individual, lo cual es de gran utilidad por la gran variedad de variables que contiene. Además, este tipo de estudios espaciales son pocos usuales, la mayoría son en establecimientos de salud, locales o departamentales, por lo tanto, la información de la ENDES, al ser más general, nos aproxima a mejores extrapolaciones de la realidad nacional que sean de utilidad para desarrollar estrategias de prevención y control de hipertensión arterial. Finalmente, estos estudios se ven afectados por extensiones geográficas y densidades de los eventos estudiados.

En conclusión, los departamentos de la costa norte, sierra norte, como Cajamarca y Amazonas y de la selva, como Loreto y Madre Dios, son aquellos que presentan conglomerados alto-alto de hipertensos. Por ello, es imperativo que se ejecuten investigaciones para identificar aquellos factores que intervienen en la elevada prevalencia de hipertensión en dichos departamentos con el fin de desarrollar políticas que favorezcan a la población.
